# Efficacy and safety of Ashwagandha (*Withania somnifera*) root extract in pregnant women: a prospective, randomized, comparative, open-label, 12-week study

**DOI:** 10.3389/fgwh.2026.1767865

**Published:** 2026-02-12

**Authors:** Ashutosh Ajgaonkar, Himanshu Tayade, Anirudha Nayak

**Affiliations:** 1 Department of Obstetrics & Gynaecology Division, Trupti Hospital, Thane West, Maharashtra, India; 2 Department of Pharmacology, D Y Patil University School of Medicine, Pune, Maharashtra, India; 3 Department of Pharmacology, D Y Patil University School of Medicine, Navi Mumbai, Maharashtra, India

**Keywords:** *Withania somnifera*, hemoglobin, pregnancy, anemia, stress and sleep

## Abstract

**Introduction:**

Pregnancy is associated with increased risk of anemia, high psychological stress, and sleep disturbances, yet safe therapeutic options remain limited. Ashwagandha (*Withania somnifera*) root extract (ARE) possesses adaptogenic and hematopoietic potential, but evidence in pregnant women is scarce. The aim of the current study is to evaluate the efficacy and safety of ARE on hematological parameters and on stress, sleep quality, and laboratory safety markers in pregnant women.

**Methods:**

This 12-week, prospective, randomized, open-label, comparative trial enrolled 70 pregnant women in the second trimester. Participants received either ARE 300 mg twice daily plus standard hematinic therapy or standard hematinic therapy (standard of care; SOC) alone. Primary endpoints included changes in hemoglobin (Hb) and red blood cell (RBC) indices. Secondary endpoints included perceived stress, sleep quality, and safety assessments (adverse events, liver, renal, cardiac, and thyroid markers). Efficacy was analyzed in the per-protocol population; safety in the intention-to-treat population.

**Results:**

Of 70 randomized participants, 63 completed the study (ARE: *n* = 32; SOC: *n* = 31). ARE supplementation produced significant improvements in hematological parameters: Hb increased by 1.06 ± 0.44 g/dL vs. 0.78 ± 0.24 g/dL with SOC (between-group difference 0.28 g/dL; *p* = 0.003), mean corpuscular hemoglobin concentration improved significantly (difference 0.73 g/dL; *p* = 0.017), and red cell distribution width decreased markedly (difference −0.57%; *p* < 0.001). RBC count and hematocrit showed favorable but non-significant trends. ARE significantly reduced perceived stress at Week 12 (–10.50 vs. −5.35; *p* < 0.001) and improved key sleep parameters, including subjective sleep quality at all visits (*p* = 0.036), sleep duration (Week 8 and 12; *p* < 0.001), and sleep disturbances (Week 12; *p* = 0.028). No adverse events or serious adverse events occurred. Laboratory evaluations showed no detrimental effects on hepatic, renal, or thyroid function.

**Discussion:**

ARE supplementation for 12 weeks improved hemoglobin levels, RBC quality, perceived stress, and multiple aspects of sleep in pregnant women, with no adverse events and stable organ-function markers. ARE appears to be a tolerable and suitable adjunct to standard prenatal care for supporting hematological and psychological well-being during pregnancy.

**Clinical Trial Registration:**

https://ctri.nic.in/Clinicaltrials/pmaindet2.php?EncHid=MTIyNjQ2&Enc=&userName=, identifier CTRI/2025/01/079238 on January 22, 2025.

## Introduction

The prenatal period is a phase of physiological and psychological change for both the mother and the fetus. This stage is indicated by rapid cellular, hormonal, and metabolic modifications that are crucial for supporting maternal well-being and optimal fetal growth (1). Several health factors like anemia, psychological stress, and sleep inadequacy require close monitoring during pregnancy, as they significantly influence the maternal and neonatal outcome (2).

Anemia is one of the predominant medical complications during pregnancy and is considered a major global public health concern. According to the World Health Organization (WHO, 2025), about 35.5% of pregnant women worldwide suffer from anemia. Although frequency has reduced from 40.3% (2000) to 33.5% (2023), anemia in pregnancy is still considered a major global health challenge (WHO global anemia estimates, 2025 (3). Maternal anemia is associated with serious complications like premature membrane rupture, postpartum hemorrhage, and impaired postpartum recovery. For the fetus, anemia increases the risk of low birth weight, preterm delivery, and neonatal asphyxia. Therefore, regular monitoring of hemoglobin (Hb) levels and timely treatment remain essential for prenatal care (4).

Along with anemia, psychological stress has developed as a serious but often underrecognized factor during pregnancy (5). Prenatal stress is associated with both maternal complications and adverse child outcomes, including altered neurodevelopment and increased susceptibility to metabolic disorders later in life (6).

Stress not only affects as a single factor but also contributes to anemia through mechanisms such as reduced nutrient absorption (iron and folate), increased hepcidin activity, systemic inflammation, and oxidative damage to erythrocytes (7). Sleep disturbance represents another interlinked factor, where stress disrupts sleep quality, while poor sleep further aggravates stress and fatigue (8). Nutrient deficiencies, including iron and magnesium depletion during chronic stress, additionally impair restorative sleep, creating a triangle of interconnected health burdens during pregnancy (9).

Management of stress, insomnia, and mild mental health symptoms in pregnancy presents unique clinical challenges. While non-pharmacological strategies such as relaxation techniques, lifestyle modification, and sleep hygiene are preferred as first-line interventions, many women may require additional therapeutic support (10). However, pharmacological treatment options remain limited due to concerns about placental transfer, teratogenic potential, neonatal adaptation syndromes, and maternal tolerability. This therapeutic dilemma has led to growing interest in alternative strategies, particularly herbal medicines, which are often perceived as natural and safe (11). In addition to routine supplementation, supportive therapies may play a complementary role in enhancing maternal well-being and addressing interconnected factors such as stress, sleep disturbance, and nutritional status (12).

Women are the primary users of herbal medicines and often continue their use during pregnancy, with prevalence ranging from 7% to 55% depending on geographic location, ethnicity, cultural traditions, and social status. Reported usage includes about 34% in Australia, nearly 50% in the European Union, and 6%–9% in the USA and Canada (13). The current study focused on Ashwagandha.

Ashwagandha (*Withania somnifera*), a traditional adaptogenic herb from Ayurvedic medicine, has received increasing scientific attention for its adaptogenic, antioxidant, anti-inflammatory, and neuroprotective properties. It regulates the hypothalamic-pituitary-adrenal (HPA) axis, normalizes cortisol levels, and exerts GABAergic and serotonergic effects, thereby promoting resilience to stress. Preliminary evidence also supports its role in enhancing cognitive performance, reducing fatigue, and improving sleep quality (14, 15). Preclinical investigations further support its safety, with orally administered Ashwagandha root extract (ARE) demonstrating a No Observed Adverse Effect Level (NOAEL) of 2,000 mg/kg body weight. Notably, no toxicity was observed in developing fetuses or pups when adult male and female rats received the extract during reproduction, highlighting its favorable safety profile in reproductive contexts (16).

Notably, a multi-herbal formulation containing *Withania somnifera*, *Emblica officinalis*, *Asparagus racemosus*, and *Pueraria tuberosa* has previously demonstrated efficacy and safety in managing anemia among pregnant women (17). However, the isolated efficacy and safety profile of Ashwagandha root extract during pregnancy has not been systematically evaluated. Given its growing popularity, elucidating its role in maternal health is both timely and essential.

In view of the need for safe and effective interventions addressing stress, anemia, and sleep disturbances during pregnancy, and the increasing use of herbal supplements in the pregnant population, the present investigation is designed to evaluate ARE as a single intervention. This study is a prospective, randomized, comparative, 12-week clinical trial aimed at rigorously assessing both the efficacy and safety of ARE in pregnant women.

## Materials and methods

### Study objectives

#### Primary objectives

The study evaluated the efficacy of ARE on Hb and red blood cell (RBC) indices in pregnant women.

#### Secondary objectives

The study evaluated the safety of ARE in pregnant women, as well as its effects on stress levels and sleep quality.

### Study design

This was an open-label, prospective, randomized, comparative, 12-week treatment period trial. The clinical study protocol was approved by the Institutional Ethics Committee (IEC) of Biomedical and Health Research (IEC Reference No: EC/NEW/INST/2019/473). The study was prospectively registered with the Clinical Trials Registry of India (CTRI) with registration number CTRI/2025/01/079238 on January 22, 2025. The study followed the principles described in the Consolidated Standards of Reporting Trials (CONSORT) declaration. This study was carried out in compliance with the ICH GCP E6 R2 2016(Step 4), ‘Guidance for Good Clinical Practices (GCP)’, New Drugs and Clinical Trials Rules (2019) and Declaration of Helsinki (Taipei 2016). Written informed consent was obtained from each pregnant woman. Investigators provided a full explanation of the protocol, which was documented on a written consent form available in Hindi, English, and Marathi (as applicable to the participant). Participants attended a screening/enrollment/baseline visit (Day 1), followed by visits at weeks 4, 8, and 12. The study was conducted at Trupti Hospital, Thane, Maharashtra, India, from 01 February 2025 to 28 May 2025.

The test group received capsules containing ARE (300 mg twice daily) as add-on therapy to the standard of care (SOC), which included an Autrin Hematinic Capsule containing Cyanocobalamin (15 mcg), Iron (98.6 mg) & Folic Acid (1.5 mg) twice daily. The control group received the standard hematinic only. The enrolled participants self-administered the oral intervention and were asked to return any unused dosages at Visit 4 and Week 12. Adherence to the assigned regimen was assessed by study personnel by recording the number of capsules dispensed and returned at each visit. Subjects who took 80% or more of the planned study medications were considered compliant.

### Randomization

The randomization schedule was generated using the PC-based program Rando (Version 1.2, R. Raveendran). After providing written informed consent, each subject was assigned a unique screening number, and upon confirmation of eligibility, a randomization number was allocated according to the schedule. In this open label trial, subjects were randomized in a 1:1 ratio to receive either the intervention or the comparator.

### Sample size

A total of 70 healthy pregnant women were enrolled to assess the efficacy and safety of standardized Ashwagandha root extract. Sample size was calculated based on effect sizes reported in a recent study of the Ayurvedic preparation Sangfer (BPRL, Bengaluru, India), which demonstrated an increase in Hb from 8.08 ± 0.39 to 11.19 ± 0.67 g/dL over 12 weeks, corresponding to a mean change of 3.11 g/dL (17). For the present trial, a change of 2.48 g/dL (80% of 3.11) was assumed for the standard of care (SOC) group. With a two-sided *α* of 0.05, 90% power, an expected mean difference of 0.62 g/dL between groups, and a standard deviation of 0.66, the required sample size was estimated at 56 participants (28 per arm). To assess the study objective at 80% power and 5% alpha, and allowing for a 10% dropout rate, the total planned enrollment was 70 participants (35 per arm).

### Participants

#### Inclusion criteria

Pregnant women aged 18–35 years in the second trimester (12–24 weeks) with a normal pregnancy and no significant comorbidities were enrolled. Those willing to comply with the study protocol, attend all follow-up visits, and who provided written informed consent after understanding the study procedures, risks, and benefits were included.

#### Exclusion criteria

Women with any comorbid conditions, including uncontrolled diabetes, hypertension, renal failure, or cardiovascular disease, were excluded. Those with a history of miscarriage, preterm labor, or pregnancy complications such as preeclampsia or gestational diabetes were not included. Participants with thyroid or other endocrine disorders, psychiatric disorders, substance abuse, severe anemia (Hb ≤ 8 g/dL), bleeding disorders, or life-threatening pregnancy complications within the past six months (e.g., stroke, myocardial infarction) were excluded. Women with infections or immunodeficiency disorders (e.g., HIV/AIDS or other sexually transmitted diseases), high alcohol consumption (>2 standard drinks/day), smoking, illicit drug use, or participation in another clinical trial within 30 days prior to enrollment were also excluded.

Participants were withdrawn prior to study completion in cases of voluntary withdrawal of consent, violation of the exercise or therapy regimen, occurrence of serious adverse events (SAEs), worsening of pre-existing conditions, or any other situation deemed unsafe by the investigator. All reasons for withdrawal were documented, appropriate referrals were provided, and end-of-study procedures were performed in cases of premature withdrawal.

### Investigational products

The test product is a standardized root extract of *Withania somnifera*, supplied by Ixoreal Biomed Inc. (Los Angeles, California (KSM-66 Ashwagandha ®), USA) and manufactured using a green-chemistry, aqueous-based extraction process. The extract is a light yellowish-brown, slightly hygroscopic powder, soluble in water, with a measured pH of 4.76 (5% w/v aqueous solution) and a bulk density of 0.58 g/cc (tapped bulk density 0.78 g/cc). It is standardized to contain >5% total withanolides. Withanone and withaferin A are in negligible amounts. HPLC analysis confirmed 5.33% total withanolides. Heavy metals (Pb < 0.05 ppm, Cd < 0.01 ppm, As < 0.01 ppm, Hg < 0.01 ppm) were below quantification limits. Microbial counts complied with USP 〈561〉 limits, with the absence of *Escherichia coli, Salmonella spp*., and *Staphylococcus aureus*. It is stable for three years when stored under recommended temperature conditions (22 ± 3 °C). The control group received oral hematinic Autrin Capsule containing Cyanocobalamin (15 mcg), Iron (98.6 mg) & Folic Acid (1.5 mg) twice daily.

### Study endpoints

#### Primary endpoints

##### Hb and RBC indices

The primary endpoints of the study were Hemoglobin (Hb) concentration and red blood cell (RBC) indices, including RBC count, hematocrit (HCT), mean corpuscular volume (MCV), mean corpuscular hemoglobin (MCH), mean corpuscular hemoglobin concentration (MCHC), and red cell distribution width (RDW), assessed at baseline and each follow-up visit (Weeks 4, 8 and 12).

#### Secondary endpoints

##### Clinical adverse events

Subjects were monitored continuously for adverse events (AEs), whether spontaneously reported by participants or observed by the investigator, which were recorded, managed, and assessed for intensity and causality. Intensity was rated on a three-point scale as mild (awareness of symptoms without disruption of usual activity), moderate (sufficient to affect usual activity), or severe (inability to perform usual activities). Causality was assessed on a five-point scale as unrelated, unlikely, possible, probable, or certain, based on association with the investigational product. AEs requiring therapy were treated according to recognized medical standards, with resuscitation equipment and medicines available for emergencies, and outcomes were classified as complete recovery, incomplete recovery, recovered with sequelae, unknown, or death.

SAEs were defined as events resulting in death, life-threatening situations, hospitalization or prolonged hospitalization, persistent or significant disability, or congenital anomalies. Unexpected AEs were those inconsistent with product information. Other significant AEs included marked laboratory abnormalities or events leading to interventions such as dose reduction, treatment withdrawal, or additional therapy. Participants who discontinued due to AEs were followed until resolution or stabilization, and clinically relevant laboratory abnormalities were repeated and monitored until normalized or explained. All SAEs were reported to the investigator within 24 h and documented in detail on the Serious Adverse Event Report Form.

Safety data were periodically reviewed by an independent oversight committee (clinician, statistician, ethicist), fulfilling the role of a Data Safety Monitoring Board.

### Laboratory assessments

At each scheduled prenatal visit, venous blood samples were collected from pregnant participants in the morning hours (08:00–10:00 AM) by trained obstetric nursing staff or certified phlebotomists using aseptic precautions. Approximately 3–5 mL of maternal venous blood was drawn from the antecubital vein into EDTA vacutainer tubes, ensuring minimal discomfort and adherence to safe sampling practices for pregnancy. Samples were gently inverted to maintain anticoagulation and transported promptly to the laboratory, typically within one hour. Hematological and laboratory parameters were analyzed using an automated hematology analyzer operating on impedance and photometric principles (Sysmex XN-1000), with calibration performed as per manufacturer recommendations and daily internal quality controls to ensure analytical reliability. All laboratory results were cross-verified by two independent technicians prior to entry into the study database.

### Maternal stress

The Perceived Stress Scale (PSS-10) is a widely used instrument for assessing perceived stress, measuring the degree to which individuals appraise their lives as unpredictable, uncontrollable, or overloaded. Participants reported how often they experienced specific feelings and thoughts during the past month using a 5-point scale where 0 was never, 1 was almost never, 2 was sometimes, 3 was often, and 4 was very often. Scores for items 4, 5, 7, and 8 were reverse-coded with 0 as 4, 1 as 3, 2 as 2, 3 as 1, and 4 as 0, and the total score was calculated by summing all items. Total scores were interpreted with 0–13 indicating low stress, 14–26 indicating moderate stress, and 27–40 indicating high stress (18).

### Maternal sleep quality

The Pittsburgh Sleep Quality Index (PSQI) is a validated instrument used to assess sleep quality and patterns in adults. It differentiates poor from good sleep quality by evaluating seven components, which include subjective sleep quality, sleep latency, sleep duration, habitual sleep efficiency, sleep disturbances, use of sleeping medications, and daytime dysfunction over the past month. The sum of the seven component scores produced a global score ranging from 0 to 21, with higher scores indicating worse sleep quality (19).

### Statistical analysis

Continuous variables such as age, weight, BMI, and other demographic characteristics were summarized using descriptive statistics, including the number of observations, mean and standard deviation with 95% confidence intervals for normally distributed data, or median with range for non-normally distributed data. Categorical variables, including gender and clinical examination findings, were summarized using frequencies and percentages. Medical history and physical examination results were also presented as frequencies and percentages for the safety population. Comparisons of baseline demographic data and follow-up changes between groups were performed using the student's *t*-test for continuous variables and the Chi-square test for categorical variables. Microsoft Windows-based program MedCalc® Statistical Software version 23.3.7 (MedCalc Software Ltd, Ostend, Belgium; https://www.medcalc.org; 2025) was used for analysis. All analyses were done using two-side tests at alpha 0.05 (95% confidence levels).

## Results

**Figure 1:**
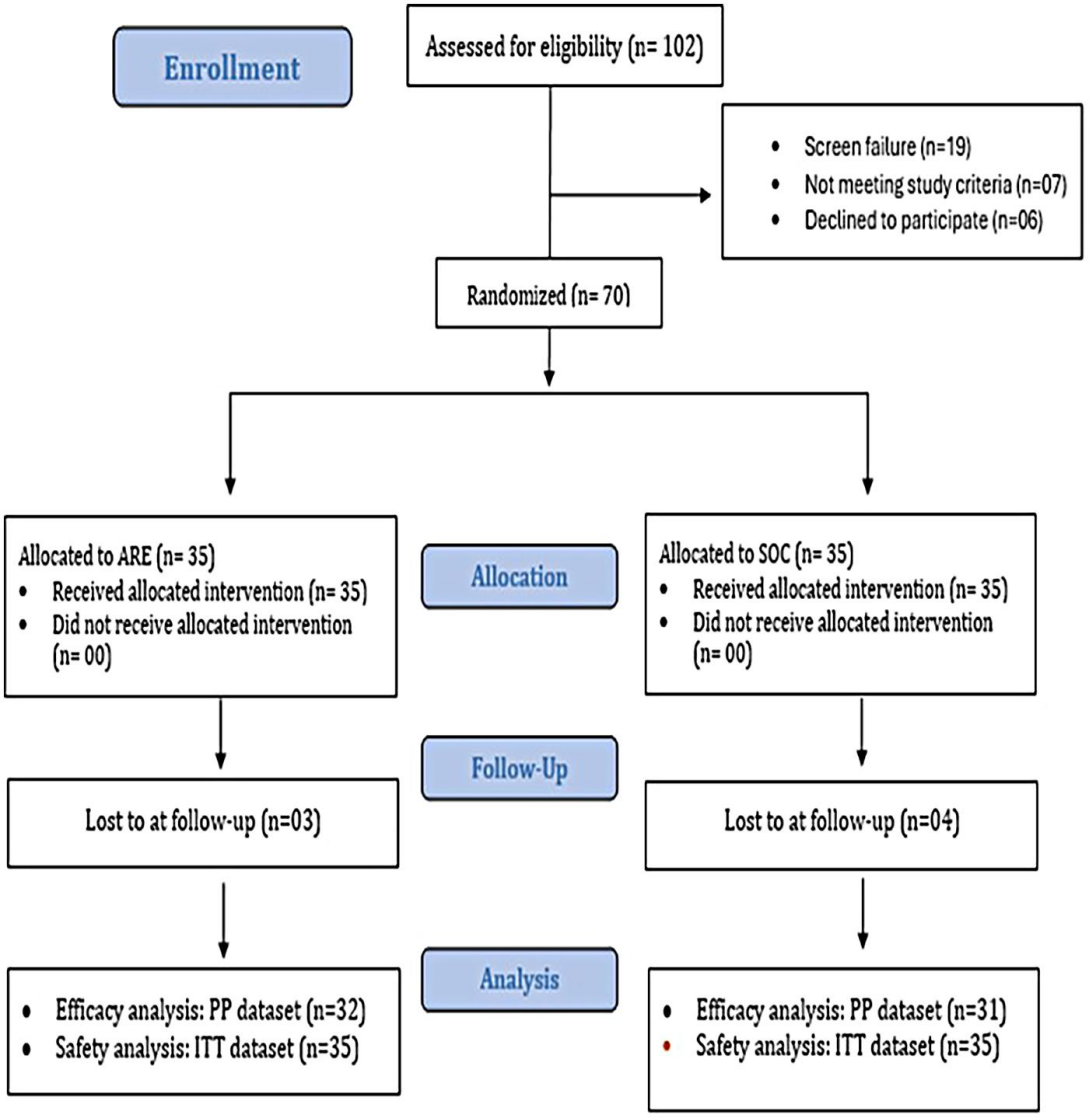
CONSORT flow diagram.

A total of 102 pregnant women were assessed for eligibility, of whom 70 met the inclusion criteria and were randomized to receive either ARE supplementation (*n* = 35) or SOC (*n* = 35) (Figure 1; Supplementary Table S1). During the study, three participants in the ARE group and four in the SOC group were lost to follow-up, resulting in 32 and 31 participants, respectively, included in the per-protocol (PP) efficacy analysis. Safety analyses were conducted on the intent-to-treat (ITT) population, which included all randomized participants (ARE: *n* = 35; SOC: *n* = 35).

At baseline, the hematological parameters, including Hb, RBC count, haematocrit, MCHC, MCV, and RDW, were comparable in the ARE and SOC groups (Table 1).

**Table 1:** Profile of participants at baseline in ITT dataset (*n* = 70).

	Parameters	ARE (*n* = 35)	SOC (*n* = 35)	Unpaired *t* test
		Mean (SD.)	Mean (SD.)	p*
Demography	Age (yrs)	29.09 (3.04)	29.83 (2.80)	0.292
	Height (cm)	157.46 (5.72)	156.11 (4.66)	0.285
	Weight (kg)	64.79 (7.36)	62.81 (7.98)	0.286
	BMI (kg/m^2^)	26.24 (3.60)	25.86 (3.75)	0.666
Physical examination	SBP (mmHg)	123.66 (6.95)	124.34 (8.01)	0.703
	DBP (mmHg)	79.31 (6.47)	80.57 (7.12)	0.442
	PR (bpm)	76.91 (5.43)	76.17 (4.40)	0.532
	Temp (°C)	98.36 (0.51)	98.23 (0.61)	0.353
	RR (breaths/min)	18.63 (2.60)	17.31 (1.75)	0.016
Haematology	Hb (g/dL)	11.55 (1.05)	11.58 (1.06)	0.909
	RBC (million/µL)	3.62 (0.36)	3.56 (0.36)	0.486
	Hct (%)	35.09 (1.26)	35.73 (1.36)	0.045
	MCV (fL)	92.02 (6.26)	89.61 (6.49)	0.118
	MCHC (g/dL)	34.34 (1.17)	34.60 (1.44)	0.410
	RDW (%)	13.63 (1.07)	13.47 (1.02)	0.528
Liver Function Test (LFT)	Total Bilirubin (mg/dL)	0.41 (0.05)	0.43 (0.04)	0.035
	Direct Bilirubin (mg/dL)	0.07 (0.04)	0.08 (0.05)	0.457
	Indirect Bilirubin (mg/dL)	0.28 (0.09)	0.28 (0.07)	0.780
	ALT (U/L)	10.12 (3.89)	10.36 (3.89)	0.804
	AST (U/L)	14.63 (2.60)	14.46 (2.60)	0.803
	ALP (U/L)	94.68 (29.23)	96.21 (24.94)	0.814
Renal Function Test (RFT)	Creatinine (mg/dL)	0.55 (0.25)	0.53 (0.28)	0.828
	BUN (mg/dL)	8.61 (2.16)	8.46 (2.09)	0.776
Cardiac Marker	CK-MB (ng/mL)	3.00 (0.68)	3.13 (0.76)	0.459
Thyroid profile	TSH (µIU/mL)	1.42 (0.94)	1.32 (0.95)	0.640
	T4 (µg/dL)	9.18 (6.78)	11.12 (6.85)	0.237
	T3 (ng/dL)	120.46 (59.55)	136.41 (59.99)	0.268
Perceived Stress Score (PSS)	PSS (score)	18.31 (2.92)	18.17 (1.79)	0.806
Pittsburgh Sleep Quality Index (PSQI) Domain Score**	Time to sleep	1.17 (0.86)	1.09 (0.82)	0.670
	Sleep duration (hrs.)	7.43 (1.39)	7.24 (1.39)	0.579
	Sleep quality (C1; Sub)	2.66 (0.48)	2.57 (0.56)	0.494
	Sleep latency (C2)	2.00 (1.00)	2.09 (0.82)	0.696
	Sleep duration (C3)	1.77 (1.31)	1.80 (1.23)	0.925
	Sleep efficiency (C4)	1.37 (1.35)	1.26 (1.12)	0.701
	Sleep disturbance (C5)	2.49 (0.56)	2.51 (0.56)	0.832
	Sleep medication (C6)	1.71 (0.71)	1.89 (0.83)	0.357
	Daytime dysfunction (C7)	1.80 (0.76)	1.77 (1.14)	0.902
	Global PSQI score	13.80 (2.81)	13.89 (2.26)	0.888

* *p*, value was obtained using an unpaired *t* test; ITT, intent-to-treat; ARE, Ashwagandha root extract; SOC, standard of care; Age (yrs.), age in years; BMI (kg/sq.m^2^), body mass index in kilograms per square meter; DBP, diastolic blood pressure; PSQI, Pittsburgh sleep quality index; No., number; hrs., hours; min., minutes; SBP, systolic blood pressure; mmHg, millimeters of mercury.

** Component-wise Scoring of Sleep Parameters (C1–C7): Sleep Duration (hours: Total hours of actual sleep per night (e.g., 9 h). Score is derived from duration as reported.

C1: Subjective Sleep Quality Score; C2: Sleep latency score based on time to fall asleep, ≤15 min = 0; 16–30 min = 1; 31–60 min = 2; >60 min = 3; C3: Sleep Disturbance Score—Based on #4 score (number of actual sleep hours), >7 h = 0; 6–7 h = 1; 5–6 h = 2; <5 h = 3; C4: Sleep Efficiency (Total hours asleep ÷ Total hours in bed) × 100 Scoring, >85% = 0; 75%–84% = 1; 65%; 74% = 2; <65% = 3; C5: Sleep Disturbances Composite Score; Sum of scores from items #5b to #5j, 0 = 0; 1–9 = 1; 10–18 = 2; 19 27 = 3; C6: Use of Sleep Medications; Score derived directly from item #6; C7: Daytime Dysfunction Score, Sum of scores from items #7 and #8: 0 = 0; 1–2 = 1; 3–4 = 2; 5–6 = 3.

### Hb and RBC indices

**Figure 2:**
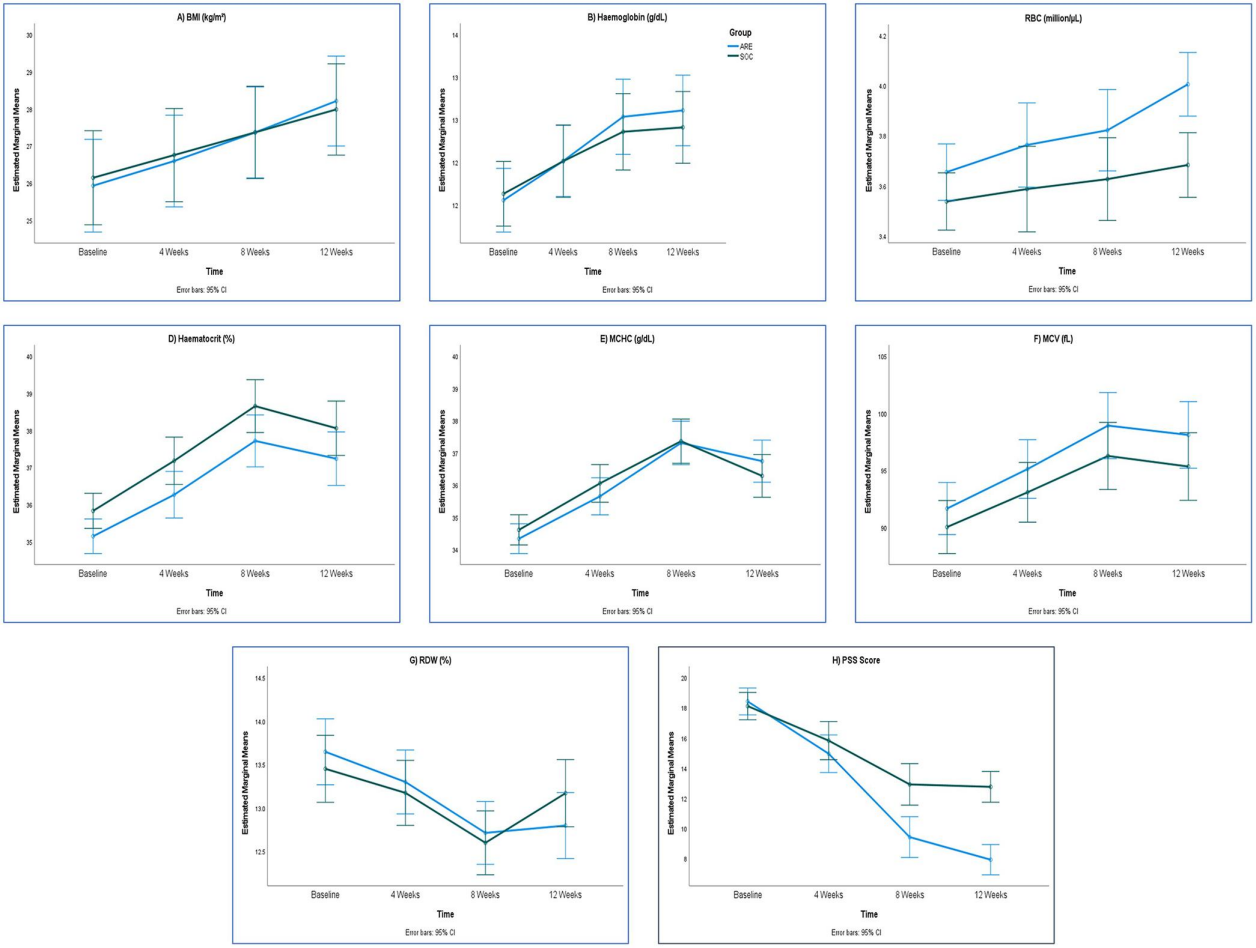
BMI and hematological laboratory parameters and PSS score in PP dataset (*n* = 63). ARE, Ashwagandha root extract; BMI, body mass index; MCV, mean corpuscular volume; MCHC, mean corpuscular hemoglobin concentration; PSS Score, perceived stress scale score; RBC, red blood cells; RDW, red cell distribution width; SOC, standard of care. **(A)**: BMI; **(B)**: Hemoglobin; **(C)**: RBC; **(D)**: Hematocrit; **(E)**: MCHC; **(F)**: MCV; **(G)**: RDW; **(H)**: PSS Score.

Table 2 presents that over the 12-week study period, ARE supplementation produced significantly greater increases in Hb in Week 8 and Week 12 (*p* = 0.014 and *p* = 0.003, respectively), with moderate to large effect sizes. Similarly, MCHC showed a significant improvement in the ARE group at Week 12 (*p* = 0.017), while RDW demonstrated a marked reduction compared to SOC at Week 12 (*p* < 0.001, Cohen's d = –1.216), indicating a large effect. RBC count, haematocrit changes, and MCV increases did not differ significantly between groups, though a trend toward higher RBC values was observed in the ARE arm at Week 12 (*p* = 0.088). Figure 2 illustrates the hematological laboratory parameters in PP dataset (*n* = 63).

**Table 2:** Hematological laboratory parameter in PP dataset (*n* = 63).

Parameters	ARE (*n* = 32)	SOC (*n* = 31)	Difference	Unpaired *t* test		Effect Size
	Mean (SD.)	Mean (SD.)	Mean (95% C.I.)	“*t*”	p*	*Cohen's* “*d*”
Haemoglobin (g/dL)						
Baseline	11.55 (1.03)	11.63 (1.09)	−0.08 (−0.61 to 0.46)	−0.284	0.777	−0.072
Change at Week 4	0.46 (0.27)	0.38 (0.31)	0.08 (−0.07 to 0.22)	1.083	0.283	0.273
Change at Week 8	0.98 (0.43)	0.73 (0.37)	0.25 (0.05 to 0.45)	2.517	0.014	0.634
Change at Week 12	1.06 (0.44)	0.78 (0.24)	0.28 (0.1 to 0.46)	3.054	0.003	0.77
RBC (million/µL)						
Baseline	3.65 (0.34)	3.54 (0.29)	0.12 (−0.04 to 0.28)	1.461	0.149	0.368
Change at Week 4	0.11 (0.38)	0.05 (0.60)	0.06 (−0.19 to 0.31)	0.464	0.644	0.117
Change at Week 8	0.17 (0.32)	0.09 (0.61)	0.08 (−0.16 to 0.32)	0.644	0.522	0.162
Change at Week 12	0.35 (0.45)	0.15 (0.49)	0.21 (−0.03 to 0.44)	1.731	0.088	0.436
Haematocrit (%)						
Baseline	35.14 (1.28)	35.82 (1.36)	−0.69 (−1.35 to −0.02)	−2.061	0.044	−0.519
Change at Week 4	1.12 (0.94)	1.35 (1.01)	−0.23 (−0.72 to 0.26)	−0.943	0.349	−0.238
Change at Week 8	2.57 (1.36)	2.83 (1.40)	−0.25 (−0.95 to 0.44)	−0.726	0.471	−0.183
Change at Week 12	2.09 (1.42)	2.23 (1.32)	−0.14 (−0.83 to 0.55)	−0.394	0.695	−0.099
MCHC (g/dL)						
Baseline	34.33 (1.16)	34.60 (1.44)	−0.27 (−0.93 to 0.38)	−0.83	0.409	−0.209
Change at Week 4	1.31 (0.89)	1.44 (1.03)	−0.13 (−0.61 to 0.35)	−0.536	0.594	−0.135
Change at Week 8	2.97 (1.31)	2.75 (1.41)	0.22 (−0.47 to 0.91)	0.639	0.525	0.161
Change at Week 12	2.41 (1.17)	1.67 (1.20)	0.73 (0.14 to 1.33)	2.454	0.017	0.619
MCV (fL)						
Baseline	91.63 (6.35)	90.01 (6.58)	1.62 (−1.64 to 4.88)	0.995	0.324	0.251
Change at Week 4	3.46 (2.40)	3.04 (2.53)	0.42 (−0.82 to 1.66)	0.676	0.502	0.17
Change at Week 8	7.27 (3.82)	6.23 (3.55)	1.04 (−0.82 to 2.9)	1.118	0.268	0.282
Change at Week 12	6.45 (3.55)	5.30 (3.70)	1.15 (−0.68 to 2.98)	1.258	0.213	0.317
RDW (%)						
Baseline	13.64 (1.10)	13.45 (1.05)	0.2 (−0.35 to 0.74)	0.723	0.472	0.182
Change at Week 4	−0.35 (0.35)	−0.28 (0.35)	−0.07 (−0.24 to 0.1)	−0.806	0.424	−0.203
Change at Week 8	−0.93 (0.52)	−0.85 (0.47)	−0.08 (−0.33 to 0.17)	−0.657	0.514	−0.165
Change at Week 12	−0.85 (0.51)	−0.28 (0.42)	−0.57 (−0.81 to −0.33)	−4.825	0.001	−1.216

* *p*, value was obtained using an unpaired *t*-test; SD., standard deviation; PP, per protocol; ARE, Ashwagandha root extract; SOC, standard of care; Hb, hemoglobin; RBC, red blood cells; Hct, hematocrit; MCV, mean corpuscular volume; MCHC, mean corpuscular hemoglobin concentration; RDW, red cell distribution width; ALT, alanine transaminase; AST, aspartate transaminase; ALP, alkaline phosphatase; BUN, blood urea nitrogen; TSH, thyroid stimulating hormone; T4, thyroxine; T3, triiodothyronine; kg, kilogram; cm, centimeter; m², square meter; g/dL, grams per deciliter; mg/dL, milligrams per deciliter; U/L, units per liter; µIU/mL, micro-international units per milliliter; µg/dL, micrograms per deciliter; ng/dL, nanograms per deciliter.

### Clinical adverse events

No adverse events were reported in any of the participants.

### Laboratory parameters

Table 3 shows the BMI and safety laboratory parameters in the PP dataset. Baseline values for BMI and biochemical parameters were similar between the ARE and SOC groups. Over the 12-week study period, BMI increased significantly more in the ARE group compared to SOC, with a mean difference of 0.44 kg/m² at Week 12 (*p* = 0.025, Cohen's d = 0.579). The observed increase in BMI should be interpreted cautiously, as gestational weight gain is expected during pregnancy and does not necessarily indicate a treatment-specific benefit. Liver function markers, including total, direct, and indirect bilirubin, ALT, and ALP, showed no significant between-group differences across time points. However, AST levels demonstrated a significantly greater reduction in the ARE group at Week 12 (*p* = 0.007, d = –0.707), indicating a moderate to large effect size. Renal function indices (creatinine, BUN) and cardiac marker CK-MB remained stable and comparable between groups throughout the study. Thyroid function tests (TSH, T4, T3) also showed no significant differences, with changes from baseline to Week 12 being minimal in both groups. Overall, ARE supplementation was associated with a significant improvement in BMI and a reduction in AST, while other biochemical parameters remained unaffected compared to SOC. Figure 2 shows the BMI parameter in the PP dataset.

**Table 3:** BMI and laboratory parameter in PP dataset (*n* = 63).

Parameters	ARE (*n* = 32)	SOC (*n* = 31)	Difference	Unpaired *t* test		Effect Size
	Mean (SD.)	Mean (SD.)	Mean (95% C.I.)	*“t”*	p*	*Cohen's “d”*
BMI (kg/m^2^)						
Baseline	25.92 (3.53)	26.13 (3.54)	−0.21 (−1.99 to 1.57)	−0.241	0.811	−0.061
Change at Week 4	0.66 (0.18)	0.61 (0.27)	0.05 (−0.06 to 0.17)	0.928	0.357	0.234
Change at Week 8	1.44 (0.32)	1.22 (0.59)	0.21 (−0.02 to 0.45)	1.793	0.078	0.452
Change at Week 12	2.28 (0.55)	1.84 (0.93)	0.44 (0.06 to 0.83)	2.296	0.025	0.579
Total Bilirubin (mg/dL)						
Baseline	0.41 (0.05)	0.43 (0.04)	−0.02 (−0.04 to 0.00)	−1.916	0.06	−0.483
12 Weeks	0.50 (0.09)	0.51 (0.08)	−0.01 (−0.06 to 0.03)	−0.611	0.543	−0.154
Change at Week 12	0.09 (0.08)	0.08 (0.08)	0.01 (−0.03 to 0.05)	0.436	0.665	0.11
Direct Bilirubin (mg/dL)						
Baseline	0.07 (0.04)	0.07 (0.05)	−0.01 (−0.03 to 0.02)	−0.482	0.632	−0.121
12 Weeks	0.14 (0.01)	0.14 (0.03)	−0.01 (−0.02 to 0.00)	−1.142	0.258	−0.288
Change at Week 12	0.07 (0.05)	0.07 (0.06)	0.00 (−0.03 to 0.03)	−0.086	0.932	−0.022
Indirect Bilirubin (mg/dL)						
Baseline	0.27 (0.09)	0.28 (0.07)	−0.01 (−0.05 to 0.03)	−0.469	0.64	−0.118
12 Weeks	0.36 (0.09)	0.37 (0.07)	−0.01 (−0.05 to 0.03)	−0.306	0.76	−0.077
Change at Week 12	0.09 (0.05)	0.09 (0.06)	0.00 (−0.02 to 0.03)	0.27	0.788	0.068
ALT (U/L)						
Baseline	10.20 (4.05)	10.46 (3.82)	−0.27 (−2.25 to 1.72)	−0.27	0.788	−0.068
12 Weeks	9.20 (3.94)	10.37 (2.04)	−1.17 (−2.76 to 0.42)	−1.472	0.146	−0.371
Change at Week 12	−0.99 (1.44)	−0.09 (3.22)	−0.90 (−2.15 to 0.35)	−1.442	0.154	−0.363
AST (U/L)						
Baseline	14.88 (2.57)	14.27 (2.81)	0.61 (−0.74 to 1.97)	0.906	0.369	0.228
12 Weeks	14.08 (2.43)	14.16 (3.01)	−0.09 (−1.46 to 1.29)	−0.126	0.9	−0.032
Change at Week 12	−0.81 (1.04)	−0.11 (0.94)	−0.70 (−1.20 to −0.20)	−2.804	0.007	−0.707
ALP (U/L)						
Baseline	94.74 (28.50)	97.73 (25.62)	−2.99 (−16.66 to 10.68)	−0.438	0.663	−0.11
12 Weeks	96.40 (29.30)	98.93 (25.38)	−2.53 (−16.36 to 11.30)	−0.366	0.716	−0.092
Change at Week 12	1.66 (2.61)	1.20 (3.02)	0.46 (−0.96 to 1.88)	0.65	0.518	0.164
Creatinine (mg/dL)						
Baseline	0.56 (0.24)	0.52 (0.28)	0.04 (−0.10 to 0.17)	0.562	0.576	0.142
12 Weeks	0.57 (0.22)	0.56 (0.27)	0.02 (−0.11 to 0.14)	0.267	0.79	0.067
Change at Week 12	0.02 (0.04)	0.04 (0.08)	−0.02 (−0.05 to 0.01)	−1.308	0.196	−0.33
BUN (mg/dL)						
Baseline	8.54 (2.21)	8.57 (2.05)	−0.03 (−1.11 to 1.04)	−0.06	0.952	−0.015
12 Weeks	8.72 (1.71)	8.67 (2.58)	0.05 (−1.05 to 1.15)	0.092	0.927	0.023
Change at Week 12	0.18 (0.85)	0.10 (2.96)	0.08 (−1.01 to 1.17)	0.151	0.88	0.038
CK-MB (ng/mL)						
Baseline	3.00 (0.71)	3.13 (0.79)	−0.13 (−0.51 to 0.24)	−0.715	0.477	−0.18
12 Weeks	3.04 (0.74)	3.20 (0.82)	−0.16 (−0.56 to 0.23)	−0.822	0.414	−0.207
Change at Week 12	0.04 (0.09)	0.07 (0.07)	−0.03 (−0.07 to 0.01)	−1.369	0.176	−0.345
TSH (µIU/mL)						
Baseline	1.39 (0.92)	1.23 (0.89)	0.17 (−0.29 to 0.62)	0.73	0.468	0.184
12 Weeks	1.41 (0.94)	1.24 (0.90)	0.18 (−0.29 to 0.64)	0.756	0.453	0.19
Change at Week 12	0.02 (0.05)	0.01 (0.03)	0.01 (−0.01 to 0.03)	0.895	0.374	0.226
T4 (µg/dL)						
Baseline	9.36 (6.92)	11.19 (6.86)	−1.83 (−5.30 to 1.65)	−1.052	0.297	−0.265
12 Weeks	9.58 (7.05)	11.39 (6.99)	−1.81 (−5.35 to 1.73)	−1.023	0.31	−0.258
Change at Week 12	0.22 (0.34)	0.20 (0.25)	0.02 (−0.13 to 0.17)	0.233	0.816	0.059
T3 (ng/dL)						
Baseline	121.40 (59.89)	136.15 (59.84)	−14.74 (−44.91 to 15.42)	−0.977	0.332	−0.246
12 Weeks	123.36 (60.24)	138.09 (60.78)	−14.73 (−45.22 to 15.76)	−0.966	0.338	−0.243
Change at Week 12	1.96 (3.02)	1.94 (3.78)	0.02 (−1.70 to 1.74)	0.018	0.985	0.005

* *p*, value was obtained using an unpaired *t*-test; SD., standard deviation; PP, per protocol; ARE, Ashwagandha root extract; SOC, standard of care; BMI, body mass index; Hb, hemoglobin; RBC, red blood cells; Hct, hematocrit; MCV, mean corpuscular volume; MCHC, mean corpuscular hemoglobin concentration; RDW, red cell distribution width; ALT, alanine transaminase; AST, aspartate transaminase; ALP, alkaline phosphatase; BUN, blood urea nitrogen; TSH, thyroid stimulating hormone; T4, thyroxine; T3, triiodothyronine; kg, kilogram; cm, centimeter; m², square meter; g/dL, grams per deciliter; mg/dL, milligrams per deciliter; U/L, units per liter; µIU/mL, micro-international units per milliliter; µg/dL, micrograms per deciliter; ng/dL, nanograms per deciliter.

**Table 4:** PSS and PSQI score in in PP dataset (*n* = 63

Parameters	ARE (*n* = 32)	SOC (*n* = 31)	Difference	Unpaired *t* test		Effect Size
	Mean (SD.)	Mean (SD.)	Mean (95% C.I.)	*“t”*	p*	Cohen's “*d*”
PSS (score)						
Baseline	18.41 (3.01)	18.10 (1.89)	0.31 (−0.96 to 1.58)	0.487	0.628	0.123
Change at Week 4	−3.47 (4.32)	−2.29 (1.42)	−1.18 (−2.81 to 0.45)	−1.446	0.153	−0.364
Change at Week 8	−9.00 (5.91)	−5.19 (1.58)	−3.81 (−6.00 to −1.61)	−3.468	0.001	−0.874
Change at Week 12	−10.50 (4.22)	−5.35 (1.76)	−5.15 (−6.78 to −3.51)	−6.278	0.001	−1.582
PSQI score						
Time to sleep (min.)						
Baseline	1.16 (0.88)	1.06 (0.85)	0.09 (−0.35 to 0.53)	0.419	0.677	0.106
Change at Week 4	−0.22 (0.75)	0.16 (0.73)	−0.38 (−0.75 to −0.01)	−2.030	0.047	−0.512
Change at Week 8	−0.13 (0.83)	0.10 (0.83)	−0.22 (−0.64 to 0.20)	−1.058	0.294	−0.267
Change at Week 12	−0.13 (0.83)	0.10 (0.83)	−0.22 (−0.64 to 0.20)	−1.058	0.294	−0.267
Sleep duration (hrs.)						
Baseline	7.42 (1.45)	7.27 (1.33)	0.15 (−0.56 to 0.85)	0.420	0.676	0.106
Change at Week 4	−0.77 (2.17)	−0.05 (1.48)	−0.72 (−1.66 to 0.22)	−1.528	0.132	−0.385
Change at Week 8	−1.89 (1.80)	−0.22 (1.59)	−1.67 (−2.53 to −0.82)	−3.906	0.001	−0.984
Change at Week 12	−1.89 (1.80)	−0.22 (1.59)	−1.67 (−2.53 to −0.82)	−3.906	0.001	−0.984
Sleep quality (Subjective)						
Baseline	2.66 (0.48)	2.55 (0.57)	0.11 (−0.16 to 0.37)	0.813	0.419	0.205
Change at Week 4	−0.56 (0.84)	0.13 (0.62)	−0.69 (−1.06 to −0.32)	−3.711	0.000	−0.935
Change at Week 8	−0.75 (1.16)	−0.13 (0.92)	−0.62 (−1.15 to −0.09)	−2.343	0.022	−0.590
Change at Week 12	−0.88 (1.07)	−0.35 (0.84)	−0.52 (−1.01 to −0.03)	−2.143	0.036	−0.540
Sleep latency						
Baseline	2.00 (1.02)	2.10 (0.87)	−0.10 (−0.57 to 0.38)	−0.405	0.687	−0.102
Change at Week 4	−0.56 (1.44)	−0.06 (1.09)	−0.50 (−1.14 to 0.15)	−1.545	0.127	−0.389
Change at Week 8	−0.56 (1.50)	−0.13 (1.18)	−0.43 (−1.11 to 0.25)	−1.273	0.208	−0.321
Change at Week 12	−0.75 (1.27)	−0.26 (1.12)	−0.49 (−1.10 to 0.11)	−1.626	0.109	−0.410
Sleep duration						
Baseline	1.72 (1.33)	1.81 (1.22)	−0.09 (−0.73 to 0.56)	−0.273	0.786	−0.069
Change at Week 4	0.00 (1.39)	−0.16 (1.63)	0.16 (−0.60 to 0.93)	0.422	0.674	0.106
Change at Week 8	−0.25 (1.61)	−0.03 (1.64)	−0.22 (−1.04 to 0.60)	−0.532	0.597	−0.134
Change at Week 12	−0.25 (1.61)	−0.03 (1.64)	−0.22 (−1.04 to 0.60)	−0.532	0.597	−0.134
Sleep efficiency						
Baseline	1.44 (1.37)	1.29 (1.10)	0.15 (−0.48 to 0.77)	0.470	0.640	0.118
Change at Week 4	0.19 (1.96)	0.06 (1.29)	0.12 (−0.72 to 0.96)	0.293	0.770	0.074
Change at Week 8	0.84 (1.61)	0.16 (1.13)	0.68 (−0.02 to 1.38)	1.944	0.057	0.490
Change at Week 12	0.38 (1.68)	0.10 (1.14)	0.28 (−0.45 to 1.00)	0.768	0.446	0.193
Sleep disturbance						
Baseline	2.50 (0.57)	2.55 (0.51)	−0.05 (−0.32 to 0.22)	−0.357	0.723	−0.090
Change at Week 4	−0.31 (0.74)	−0.06 (0.89)	−0.25 (−0.66 to 0.16)	−1.204	0.233	−0.303
Change at Week 8	−0.66 (0.94)	−0.23 (0.76)	−0.43 (−0.86 to 0.00)	−1.997	0.050	−0.503
Change at Week 12	−0.84 (0.92)	−0.39 (0.67)	−0.46 (−0.86 to −0.05)	−2.250	0.028	−0.567
Sleep medication						
Baseline	1.69 (0.74)	1.90 (0.87)	−0.22 (−0.62 to 0.19)	−1.063	0.292	−0.268
Change at Week 4	−0.41 (0.87)	0.00 (1.15)	−0.41 (−0.92 to 0.11)	−1.577	0.120	−0.397
Change at Week 8	−0.59 (1.39)	−0.06 (1.21)	−0.53 (−1.19 to 0.13)	−1.612	0.112	−0.406
Change at Week 12	−0.72 (1.28)	−0.13 (1.12)	−0.59 (−1.19 to 0.02)	−1.949	0.056	−0.491
Daytime dysfunction						
Baseline	1.84 (0.77)	1.71 (1.10)	0.13 (−0.34 to 0.61)	0.562	0.576	0.142
Change at Week 4	−0.34 (0.87)	0.00 (1.06)	−0.34 (−0.83 to 0.14)	−1.408	0.164	−0.355
Change at Week 8	−0.81 (0.90)	−0.10 (1.37)	−0.72 (−1.30 to −0.13)	−2.456	0.017	−0.619
Change at Week 12	−0.81 (0.90)	−0.10 (1.37)	−0.72 (−1.30 to −0.13)	−2.456	0.017	−0.619
Global PSQI score						
Baseline	13.84 (2.83)	13.90 (2.17)	−0.06 (−1.33 to 1.21)	−0.093	0.926	−0.024
Change at Week 4	−2.00 (3.60)	−0.10 (2.88)	−1.90 (−3.55 to −0.26)	−2.312	0.024	−0.583
Change at Week 8	−2.78 (3.95)	−0.52 (2.49)	−2.27 (−3.93 to −0.60)	−2.714	0.009	−0.684
Change at Week 12	−3.88 (3.88)	−1.16 (2.46)	−2.71 (−4.36 to −1.07)	−3.300	0.002	−0.832

* *p*, value was obtained using an unpaired *t*-test; CI, confidence interval; SD., standard deviation; PP, per protocol; ARE, Ashwagandha root extract; SOC, standard of care; PSS, perceived stress score; PSQI, Pittsburgh Sleep Quality Index.

### Maternal stress

Table 4 shows the stress levels over a 12-week period. Baseline PSS scores were comparable between the ARE and SOC groups (18.41 ± 3.01 vs. 18.10 ± 1.89; *p* = 0.628). At Week 4, both groups showed reductions in stress scores, with a greater though non-significant decrease in the ARE group (*p* = 0.153). By Week 8, the ARE group demonstrated a significantly larger reduction compared to SOC (–9.00 ± 5.91 vs. −5.19 ± 1.58; mean difference −3.81; 95% CI −6.00 to −1.61; *p* = 0.001; Cohen's d = –0.874). This effect was further strengthened at Week 12, where ARE supplementation resulted in a markedly greater decline in PSS scores (–10.50 ± 4.22 vs. −5.35 ± 1.76; mean difference −5.15, 95% CI −6.78 to −3.51; *p* < 0.001; Cohen's d = –1.582), indicating a large effect size.

### Maternal sleep quality

**Figure 3:**
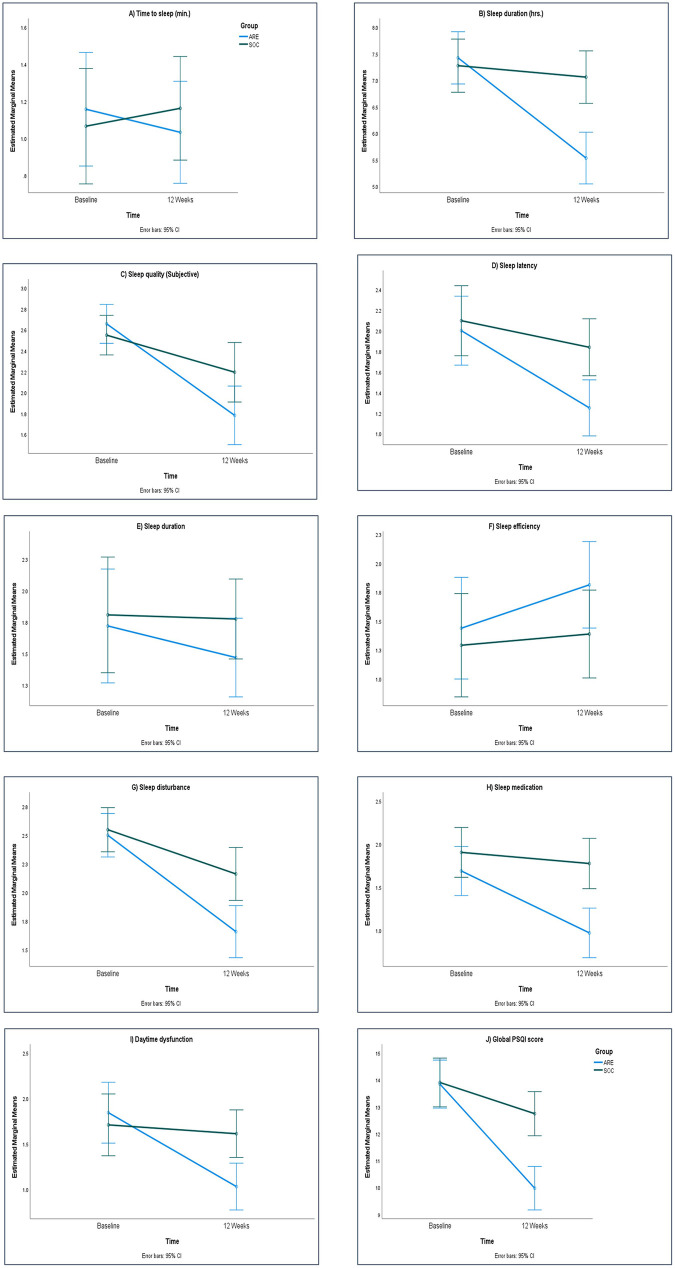
PSQI score in in PP dataset (*n* = 63). *ARE, Ashwagandha Root Extract; SOC, Standard of Care.* **(A)**: Time to Sleep; **(B)**: Sleep Duration; **(C)**: Sleep Quality; **(D)**: Sleep Latency; **(E)**: Sleep Duration; **(F)**: Sleep Efficiency; **(G)**: Sleep Disturbance; **(H)**: Sleep Medication; **(I)**: Daytime Dysfunction; **(J)**: Global PSQI Score.

At baseline, sleep quality indices measured by the PSQI were comparable between the ARE and SOC groups. Over the course of 12 weeks, participants receiving ARE demonstrated consistent improvements across several PSQI domains (Figure 3). Notably, reductions in time to sleep were evident as early as Week 4 (*p* = 0.047), while subjective sleep quality showed significant and sustained improvement at Weeks 4, 8, and 12. Sleep duration increased markedly in the ARE group, with significant differences observed at Weeks 8 and 12 (*p* < 0.001), accompanied by large effect sizes. Improvements were also seen in sleep disturbance and daytime dysfunction, both of which declined significantly in the ARE group compared to SOC at later time points. Although changes in sleep latency, sleep efficiency, and use of sleep medication favored ARE, these did not reach statistical significance. Importantly, the global PSQI score revealed robust improvements in the ARE group at Weeks 4, 8, and 12 (*p* = 0.024, 0.009, and 0.002, respectively).

## Discussion

In this 12-week study involving pregnant women, the addition of oral supplementation with Ashwagandha root extract (ARE) along with oral iron therapy resulted in significant improvements in hematological parameters, particularly Hb, MCHC, and RDW, compared with oral iron therapy alone. The observed increase in Hb and enhanced RBC quality, as reflected by improved and reduced RDW, suggests a beneficial stimulatory effect of ARE on erythropoiesis (20). These findings align with preclinical studies reporting that withanolides in ARE may enhance hematopoietic activity by promoting erythroid progenitor proliferation, modulating iron metabolism, and supporting hemoglobin synthesis, mechanisms that are particularly beneficial during pregnancy when increased blood volume and iron demands heighten susceptibility to anemia (16).

Baseline Hb levels in both groups indicate that the majority of participants had mild anemia. Though the increase in Hb observed in the ARE group at Week 12 was statistically significant compared with SOC alone; the absolute between-group difference (0.28 g/dL) was modest. Given that baseline hemoglobin values were near the lower limit of normal for pregnancy, the observed magnitude of change is likely to have limited clinical relevance at the individual level. Consequently, the hematological findings should be interpreted as supportive rather than definitive evidence of therapeutic benefit (17).

Although RBC count showed a trend toward improvement (0.21 million/µL; *p* = 0.088) with ARE, hematocrit changes with ARE were comparable to SOC, indicating that ARE primarily enhanced red cell quality rather than total blood volume expansion. The significant reduction in RDW suggests a more uniform erythrocyte population, which may translate to improved oxygen-carrying capacity and maternal-fetal health outcomes (21).

Importantly, ARE supplementation demonstrated a favorable tolerable profile across multiple organ systems. Liver enzymes were largely stable, with a modest but significant decrease in AST (–0.70 U/L; 95% CI: −1.20 to −0.20; *p* = 0.007; Cohen's d = –0.707), while ALT, ALP, and bilirubin levels remained unchanged (22). Renal markers (creatinine, BUN), cardiac marker CK-MB, and thyroid parameters (TSH, T4, and T3) showed no significant changes, supporting systemic tolerability during pregnancy (23). The absence of adverse changes in these parameters is critical in the prenatal population, reinforcing the suitability of ARE as a suitable adjunct for hematological support (24).

Overall, this study demonstrates that ARE supplementation in pregnant women effectively improves erythrocyte indices, enhances RBC quality, and is well-tolerated, suggesting a beneficial role for Ashwagandha in supporting maternal hematological health without compromising hepatic, renal, cardiac, or thyroid function. The observed reductions in perceived stress and improvements in sleep parameters among pregnant women receiving ARE are consistent with the known adaptogenic and anxiolytic properties of the herb (14, 15). Mechanistic studies indicate that withanolides, the primary bioactive constituents of ARE modulate the hypothalamic–pituitary–adrenal (HPA) axis, attenuating cortisol release and enhancing resilience to psychosocial stressors (25). By dampening HPA axis hyperactivity, ARE may reduce physiological stress responses, facilitating improvements in both perceived stress and sleep quality (14, 15). These findings are particularly reassuring given that the supplementation was administered in the second trimester, a period when maternal and fetal physiology are highly sensitive to external interventions. The absence of adverse effects confirms that ARE is tolerable when administered along with standard prenatal care.

In pregnancy, when both physiological and psychological stress can adversely affect maternal and fetal outcomes, these adaptogenic effects are particularly relevant (26). The present findings demonstrate that ARE supplementation not only lowers perceived stress scores but also improves multiple dimensions of sleep, including sleep latency, duration, and subjective quality. These improvements are likely mediated via HPA axis modulation and consequent reductions in stress-related hyperarousal, highlighting a direct mechanistic link between the biological effects of ARE and beneficial outcomes in this population (25).

Beyond stress and sleep, ARE supplementation demonstrated hematopoietic benefits, with observed improvements in erythropoiesis (27). The observed sleep benefits align with prior mechanistic studies showing that ARE promotes restorative sleep through HPA axis modulation and cortisol reduction. In the context of pregnancy, improved sleep latency and quality may translate into better maternal energy, mood regulation, and overall well-being (9, 15). Furthermore, these benefits may reduce reliance on pharmacologic sleep aids, offering a non-pharmacologic strategy for managing pregnancy-related sleep disturbances (28).

Taken together, the convergence of mechanistic evidence and clinical findings highlights the efficacy of ARE in pregnant women. By modulating the HPA axis, reducing cortisol levels, and exerting anxiolytic and adaptogenic effects, ARE supplementation may provide meaningful improvements in stress resilience, sleep quality, and hematopoietic health, without compromising maternal systemic safety (14, 15). These data support the potential role of ARE as a safe and efficacious adjunctive intervention for enhancing maternal well-being during pregnancy (17).

The absence of adverse events in this study, along with stable hepatic, renal, cardiac, and thyroid parameters, supports compliance with safety requirements typically expected for prenatal supplementation. These findings provide preliminary evidence that ARE may be integrated into maternal health strategies without violating regulatory standards.

While the present study demonstrates beneficial effects of ARE on hematological parameters, stress, and sleep in pregnant women, several limitations should be acknowledged. First, the sample size was relatively small, which may limit the generalizability of findings and the statistical power for detecting less pronounced effects. Second, the study duration was limited to 12 weeks; longer-term effects on maternal and fetal outcomes remain unknown. Third, this was a single-center study, and multi-center trials would help confirm the reproducibility of these findings across diverse populations. Fourth, fetal health outcomes were not assessed; although no maternal adverse events were reported, future studies are planned to monitor fetal development and growth parameters to ensure a comprehensive safety evaluation. The use of multiple paired and unpaired *t*-tests, rather than repeated-measures or mixed-effects modeling, represents a methodological limitation and may not fully account for within-subject correlations over time. Finally, while hematologic and sleep-related outcomes were rigorously monitored, other potential effects of ARE on metabolic or immunological parameters during pregnancy were not explored and could benefit from further investigation.

This study employed an open-label design, which introduces the potential for performance and detection bias, particularly for subjective, patient-reported outcomes such as perceived stress (PSS-10) and sleep quality (PSQI). While objective hematological and biochemical parameters are less susceptible to expectancy effects, self-reported measures may have been influenced by participants’ awareness of receiving an herbal intervention perceived as beneficial. Consequently, improvements in stress and sleep should be interpreted as associations rather than causal effects.

Given the ethical considerations in pregnancy, particularly the need to provide all participants with standard-of-care hematinic supplementation, a placebo arm was not included. Instead, participants were randomized to receive either ARE plus hematinic or hematinic alone. This design allowed evaluation of the additive benefit of ARE while ensuring no participant was deprived of essential antenatal care. The absence of a placebo-only control limits attribution of observed effects exclusively to the herbal intervention, particularly for subjective outcomes.

Routine fetal ultrasound scans were not incorporated into the study protocol, as all participants continued to receive standard antenatal care under their obstetricians. However, it was planned that in the event that an AE or SAE with potential fetal implications was reported, a fetal ultrasound scan was to be performed at the discretion of the treating physician to ensure maternal and fetal safety.

## Conclusion

In this randomized 12-week study involving pregnant women, adjunctive administration of Ashwagandha root extract (ARE) along with standard hematinic therapy was associated with modest improvements across maternal hematological parameters and patient-reported stress and sleep outcomes. The extract was well tolerated in the short term, with no clinically significant abnormalities observed in routine biochemical safety markers. Based on these findings, ARE appears to be helpful and well-tolerated, and is proposed to serve as a beneficial adjunctive option for supporting hematological health, stress resilience, and sleep quality in pregnant women.

## Data Availability

The raw data supporting the conclusions of this article will be made available by the authors, without undue reservation.
